# A first look at the reliability, validity and responsiveness of L-PF-35 dyspnea domain scores in fibrotic hypersensitivity pneumonitis

**DOI:** 10.1186/s12890-024-02991-1

**Published:** 2024-04-19

**Authors:** Jeffrey J. Swigris, Kerri Aronson, Evans R. Fernández Pérez

**Affiliations:** 1 Center for Interstitial Lung Disease, National Jewish Health, 1400 Jackson Street, G07, 80206 Denver, CO USA; 2 Division of Pulmonary and Critical Care Medicine, Weill Cornell College of Medicine, New York, NY USA

**Keywords:** Dyspnea, Hypersensitivity pneumonitis, Patient-reported outcome, Validation

## Abstract

**Background:**

Dyspnea impairs quality of life (QOL) in patients with fibrotic hypersensitivity pneumonitis (FHP). The Living with Pulmonary Fibrosis questionnaire (L-PF) assesses symptoms, their impacts and PF-related QOL in patients with any form of PF. Its scores have not undergone validation analyses in an FHP cohort.

**Methods:**

We used data from the Pirfenidone in FHP trial to examine reliability, validity and responsiveness of the L-PF-35 Dyspnea domain score (Dyspnea) and to estimate its meaningful within-patient change (MWPC) threshold for worsening. Lack of suitable anchors precluded conducting analyses for other L-PF-35 scores.

**Results:**

At baseline, Dyspnea’s internal consistency (Cronbach’s coefficient alpha) was 0.85; there were significant correlations with all four anchors (University of California San Diego Shortness of Breath Questionnaire scores *r* = 0.81, St. George’s Activity domain score *r* = 0.82, percent predicted forced vital capacity *r* = 0.37, and percent predicted diffusing capacity of the lung for carbon monoxide *r* = 0.37). Dyspnea was significantly different between anchor subgroups (e.g., lowest percent predicted forced vital capacity (FVC%) vs. highest, 33.5 ± 18.5 vs. 11.1 ± 9.8, *p* = 0.01). There were significant correlations between changes in Dyspnea and changes in anchor scores at all trial time points. Longitudinal models further confirmed responsiveness. The MWPC threshold estimate for worsening was 6.6 points (range 5–8).

**Conclusion:**

The L-PF-35 Dyspnea domain appears to possess acceptable psychometric properties for assessing dyspnea in patients with FHP. Because instrument validation is never accomplished with one study, additional research is needed to build on the foundation these analyses provide.

**Trial registration:**

The data for the analyses presented in this manuscript were generated in a trial registered on ClinicalTrials.gov; the identifier was NCT02958917.

**Supplementary Information:**

The online version contains supplementary material available at 10.1186/s12890-024-02991-1.

## Introduction

Fibrotic hypersensitivity pneumonitis (FHP) is a form of fibrosing interstitial lung disease (fILD) that, like other fILDs is incurable, induces burdensome symptoms, confers the risk of shortened survival [1, 2], and robs patients of their quality of life (QOL) [3, 4]. Although in FHP there has not been as much research into the patient experience as with idiopathic pulmonary fibrosis (IPF), available data reveal that FHP-induced dyspnea, fatigue and cough affect how patients feel and function in their daily lives [3, 4].

Given the potential for FHP to progress and respond poorly to immunosuppression and antigen avoidance (if one can be identified), Fernández Pérez and colleagues conducted a placebo-controlled trial of the antifibrotic, pirfenidone, in patients with FHP [5]. In that trial (Pirfenidone in FHP), among other patient-reported outcome measures (PROMs), the Living with Pulmonary Fibrosis (L-PF) questionnaire was used to examine the effects of pirfenidone on FHP-related QOL, symptoms and their impacts.

Here, we present findings from a hypothesis-based analysis of the reliability, validity and responsiveness of the Dyspnea domain from the 35-item L-PF (or L-PF-35; these 35 items are the same 35 that compose the Living with Idiopathic Pulmonary Fibrosis questionnaire (L-IPF) [6]).

## Methods

The design and primary results for the single-center, double-blinded Pirfenidone in FHP trial (ClinicalTrials.gov identifier NCT02958917) from which the data for our analyses were generated have been published [5]. Briefly, 40 subjects with FHP were randomized 2:1 to receive pirfenidone or a matching placebo for 52 weeks. Study visits occurred at baseline, 13, 26, 39 and 52 weeks. At each visit, subjects completed three patient-reported outcome measures (PROMs) and performed spirometry to capture forced vital capacity (FVC). Diffusing capacity (DLCO) was assessed at baseline, 26 and 52 weeks only. This analysis was performed under an approved research protocol by the National Jewish Health central Institutional Review Board (HS# 3034).

### PROMs used in the Pirfenidone in FHP trial

#### The L-PF-35 (Living with Pulmonary Fibrosis 35-Item Questionnaire)

The L-PF-35 is designed to assess PF-related QOL, symptoms and their impacts. L-PF-35 is equivalent to the Living with Idiopathic Pulmonary Fibrosis Questionnaire (L-IPF) but with the word “idiopathic” removed from the title and a single item from the Impacts Module. L-IPF began as a 44-item questionnaire, but in a previously published validation study that included 125 patients with IPF, psychometric analyses supported reducing numbers from 44 to 35 items [6]. The intent of the developer of the L-PF is to have a single, 35-item questionnaire for all forms of PF (IPF and non-IPF, including FHP). Thus, although the 44-item version (again, with the word “idiopathic” removed) was administered in the Pirfenidone in FHP trial, our analyses here were conducted on the Dyspnea domain from the 35-item version resulting from the IPF analysis. From here on, we refer to this instrument as the L-PF-35.

Percentage-of-total-possible points is used to generate the Dyspnea domain, Cough domain, Energy/Fatigue domain, and Impacts module from the L-PF-35. The Symptoms module score is derived as the average of the Dyspnea, Cough and Energy/Fatigue domain scores. The total score is the average of the Symptoms and Impacts module scores. The Symptoms module contains 15 items (Dyspnea domain 7 items, Cough domain 5 items, Energy/Fatigue domain 3 items), each with a 24-hour recall period. The Impacts module contains 20 items, each with a 7-day recall period. The range for each of the six scores is 0-100, and higher scores connote greater impairment.

#### The SGRQ (St. George’s Respiratory Questionnaire)

The SGRQ is a 50-item questionnaire that yields four scores (total, Symptoms, Activity, Impacts). For the version used in the trial, the recall period for some items is three months and for others, it is “these days”. The range for each score is 0-100, and higher scores indicate worse respiratory health status [7, 8].

#### The UCSD (University of California San Diego Shortness of Breath Questionnaire)

The UCSD is a 24-item questionnaire that assesses dyspnea severity while performing each of 21 activities, and it includes another 3 items that ask about limitations induced by shortness of breath [9]. Each item is scored on a 0–5 scale. There is no stated recall period. Scores range from 0 to 120, and higher scores indicate greater dyspnea severity.

### Statistical analyses

Baseline data were tabulated and summarized using counts, percentages and measures of central tendency. We formulated hypotheses (included in the Supplementary material) for the L-PF-35 Dyspnea domain and conducted analyses in accordance with COSMIN recommendations for studies on the measurement properties of PROMs [10, 11]. We used SGRQ Activity domain change scores, UCSD change scores, percent predicted FVC (FVC%) change, and percent predicted DLCO (DLCO%) change as anchors. Analyses included the following: (1) internal consistency and test-retest reliability, (2) convergent and known-groups analyses to assess content validity, (3) responsiveness, and (4) an estimation of the meaningful within-patient change (MWPC) threshold for worsening.

For applicable analyses, we defined worsening for the anchors in the following way: 1) ≥ 5 point increase for SGRQ Activity domain [12, 13]; 2) ≥ 5 point increase in UCSD score [14]; 3) > 2% drop in FVC% (e.g., 70% to less than 68%) [15]; and 4) ≥ 5% drop in DLCO% (e.g., 70–65% or lower). Analyses were conducted in SAS, version 9.4 (SAS Institute Inc.; Cary, NC).

#### Internal consistency

We used Cronbach’s raw coefficient alpha as the measure of internal consistency (IC). Values > 0.7 are considered acceptable.

#### Test-retest reliability

We used a two-way mixed effects model for absolute agreement to generate the intraclass correlation coefficient (ICC (2,1)) as a measure of test-retest reliability of L-PF-35 Dyspnea domain scores (from baseline to week 26) among subjects considered stable according to change (also from baseline to week 26) scores for the various anchors. Values > 0.7 are considered acceptable.

#### Convergent and known-groups validity

Convergent validity was examined using pairwise Spearman correlations between L-PF-35 Dyspnea domain scores and anchors at baseline. We used analysis of variance with secondary, p-value corrected (Tukey) pairwise comparisons to look for statistically significant differences in L-PF-35 Dyspnea domain scores between most and least severe anchor subgroup strata (with anchors di- or trichotomized based on clinically relevant cut-points; e.g., FVC: ≤55, 55 < FVC < 70, or ≥ 70).

#### Responsiveness

We used pairwise correlation, longitudinal models and empirical cumulative distribution function (eCDF) plots to assess the responsiveness of L-PF-35 Dyspnea domain scores among subjects whose dyspnea changed as defined by the applicable anchor. In the correlational analyses, for 13-, 26-, 39- and 52-week timepoints, we examined pairwise Spearman correlations between L-PF-35 Dyspnea domain change scores and anchor change. In the modeling analyses, for each anchor, we built a repeated-measures, longitudinal model with L-PF-35 Dyspnea domain change score (from baseline to each subsequent time point) as the outcome variable and anchor change (from baseline to each subsequent time point) as the lone predictor variable. Visit (week 13, 26, 39, 52) was included in each model as a class variable, and an unstructured covariance structure was used (i.e., type = un in SAS). For the eCDF, we graphed the cumulative distribution of L-PF-35 Dyspnea domain change scores from baseline to week 26 for each of two dichotomized anchor change strata (worse vs. not at week 26 as defined above).

#### Meaningful within patient change (MWPC) threshold

We used predictive modeling (anchor as the outcome and L-PF-35 Dyspnea domain as the lone predictor) and adjustment for the correlation between L-PF-35 Dyspnea domain score change and anchor score change [16] to generate MWPC threshold estimates for worsening at 26 weeks. We used the method of Trigg and Griffiths [17] to generate a correlation-weighted point estimate.

## Results

Baseline characteristics and PROM scores from the trial population are presented in Table 1. Most subjects were of non-Hispanic white ethnicity/race and supplemental oxygen users, with moderate pulmonary physiological impairment.

**Table 1:** Baseline characteristics of the 40 subjects

Variable	
Age, yrs	67.1 ± 5.7, 66.9 (63.7, 70.6)
Female(%)/Male(%)	23(57.5%) / 17 (42.5%)
Self-reported Ethnicity/Race	
Non-Hispanic White Other	37 (92.5%) 3 (7.5%)
Years since diagnosis	4.3 ± 3.9, 2.9 (1.3, 6.5)
FVC%	61.2 ± 11.9, 62.5 (55.5, 66.5)
DLCO%	52.6 ± 14.9, 50.5 (42.5, 65.5)
Supplemental Oxygen	
Any use Rest Exertion Sleep	31* 17 10 3
L-PF-35	
Total Symptoms Dyspnea Cough Fatigue Impacts	35.8 ± 18.6, 37.1 (21.0, 50.7) 31.8 ± 15.9, 32.4 (20.8, 43.3) 22.6 ± 18.5, 17.3 (9.2, 37.9) 37.5 ± 19.5, 35.0 (25.0, 50.0) 35.2 ± 22.7, 41.7(16.7, 50.0) 39.9 ± 23.2, 39.4 (18.1, 55.0)
SGRQ	
Total Symptoms Activities Impacts	45.8 ± 18.5, 49.9 (37.0, 58.2) 54.5 ± 19.4, 56.5 (40.9, 66.9) 60.6 ± 24.4, 66.5 (48.3, 79.7) 34.5 ± 19.0, 34.9 (20.3, 46.0)
UCSD SOBQ	43.5 ± 25.9, 48.5 (25.0, 63.5)

Values = mean ± standard deviation or median (interquartile limits); *1 subject missing; FVC% = percentage of the predicted forced vital capacity; DLCO% = percentage of the predicted diffusing capacity of the lung for carbon monoxide; L-PF-35 = 35-item Living with Pulmonary Fibrosis Questionnaire; SGRQ = St. George’s Respiratory Questionnaire; UCSD SOBQ = University of California San Diego Shortness of Breath Questionnaire

### Internal consistency and test-retest reliability

IC for the L-PF-35 Dyspnea domain was at least 0.85 at all time points. Test-retest reliability (TRR) coefficients for L-PF-35 Dyspnea were 0.81 or greater for each anchor. Table S1 contains IC and TRR values.

### Convergent and known-groups validity

Pairwise correlations at baseline are presented in Table 2. Correlations between L-PF-35 Dyspnea domain scores and UCSD or SGRQ Activity scores are very strong, statistically significant and in the expected directions. Correlations between L-PF-35 Dyspnea and FVC% or DLCO% are low-moderately strong, statistically significant and in the expected directions.

**Table 2:** Construct validity: Spearman correlations between L-PF-35 Dyspnea domain scores and anchors at baseline

	LPF Dyspnea Domain Score
**UCSD**	0.81 < 0.0001
**SGRQa**	0.82 < 0.0001
**FVC%**	-0.37 0.01
**DLCO%**	-0.37 0.01

Values are correlation coefficient (above) and p value (below); FVC% = percentage of the predicted forced vital capacity; DLCO% = percentage of the predicted diffusing capacity of the lung for carbon monoxide; SGRQa = SGRQ Activity score; UCSD = University of California San Diego Shortness of Breath Questionnaire

Table 3 shows results for known-groups validity analyses. For each of the four anchors, compared to the least impaired anchor subgroup, L-PF-35 Dyspnea scores were significantly worse (i.e., higher and of large effect; e.g., worse by > 1 standard deviation) for the more impaired anchor subgroup.

**Table 3:** Known-groups validity of L-PF-35 Dyspnea domain scores at baseline

Anchor Variable	N	L-PF-35 Dyspnea domain score	p
**UCSD SOBQ**			< 0.0001
≥60 30 < FVC%<60 ≤30*	13 15 12	39.2 ± 18.1 21.9 ± 12.1 5.6 ± 5.7	
**SGRQ Activity**			< 0.0001
≥60 40 < FVC%<60 ≤40*	23 11 6	32.7 ± 17.5 13.4 ± 6.8 0.7 ± 1.7	
**FVC%**			0.01
≤ 55 55 < FVC%<70 ≥70*	10 24 6	33.5 ± 18.5 20.9 ± 18.4 11.1 ± 9.8	
**DLCO%**			0.04
≤ 40 40 < FVC%<60 ≥60*	9 20 11	32.4 ± 18.7 22.0 ± 19.5 15.7 ± 14.2	
**Rest O2**			0.02
Yes No*	17 23	30.0 ± 16.1 17.2 ± 18.7	

*Reference group; FVC% = percentage of the predicted forced vital capacity; DLCO% = percentage of the predicted diffusing capacity of the lung for carbon monoxide; L-PF = 35-item Living with Pulmonary Fibrosis Questionnaire; SGRQ = St. George’s Respiratory Questionnaire; UCSD SOBQ = University of California San Diego Shortness of Breath Questionnaire

### Responsiveness

Across study timepoints, 12 of 14 correlations between L-PF-35 Dyspnea domain score change and anchor change values were statistically significant and at least moderately strong (Table S2).

**Fig. 1:**
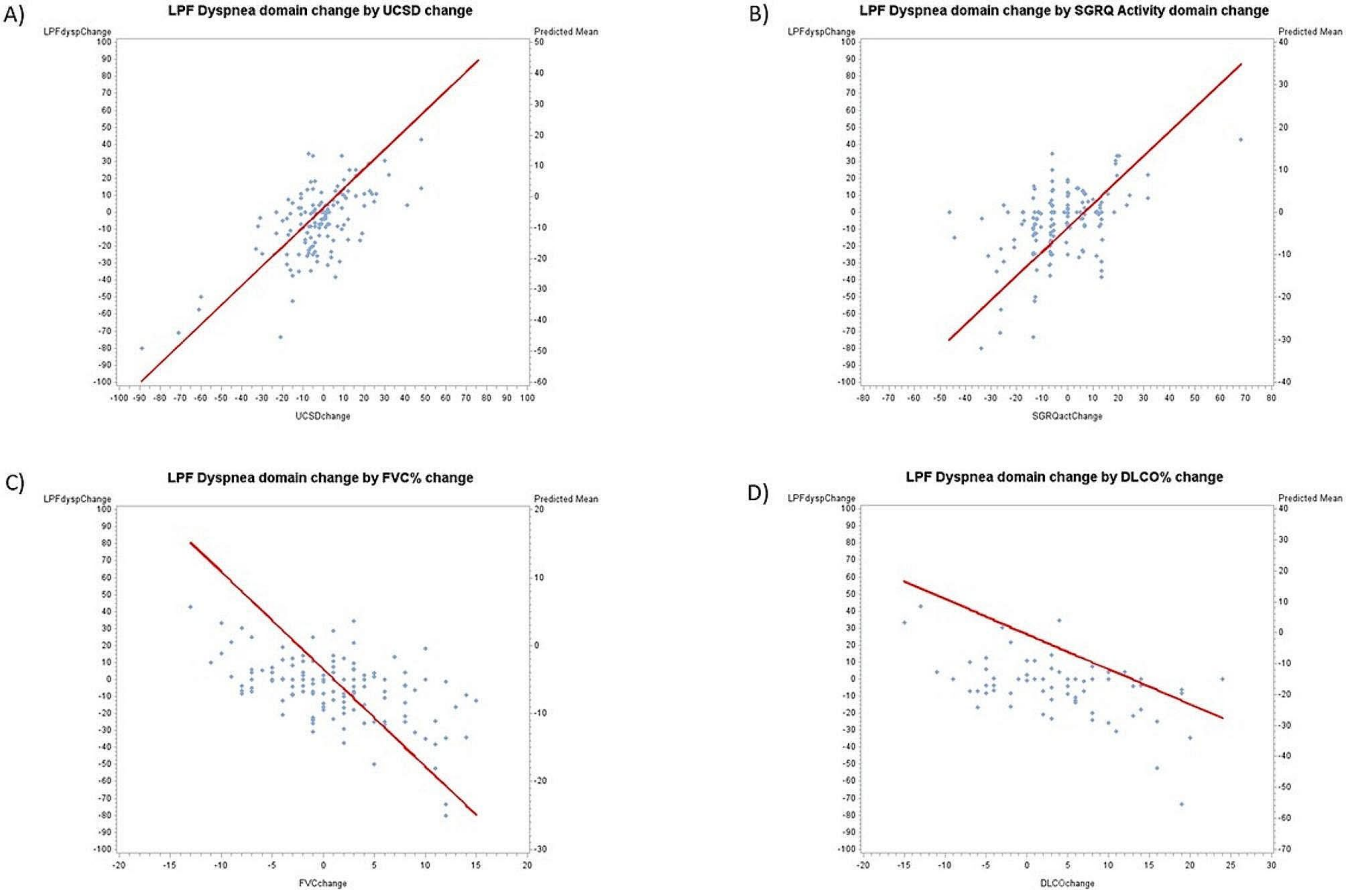
Results for mixed-effects longitudinal models showing the relationship between baseline-to-weeks 13/26/39/52 changes in L-PF-35 Dyspnea domain scores and baseline-to-weeks 13/26/39/52 changes in anchor values (Panel A: UCSD anchor, Panel B: SGRQ Activity Domain anchor, Panel C: FVC% anchor, Panel D: DLCO% anchor). Footnote: UCSD = University of California San Diego Shortness of Breath Questionnaire; SGRQ = St. George’s Respiratory Questionnaire; FVC% = percentage of the predicted forced vital capacity; DLCO% = percentage of the predicted diffusing capacity of the lung for carbon monoxide; L-PF = 35-item Living with Pulmonary Fibrosis Questionnaire

Longitudinal modeling showed significant (*p* < 0.0001 for all) associations between L-PF-35 Dyspnea domain score change and anchor change values over the course of the trial (Fig. 1). Table S3 shows results for all longitudinal models.

**Fig. 2:**
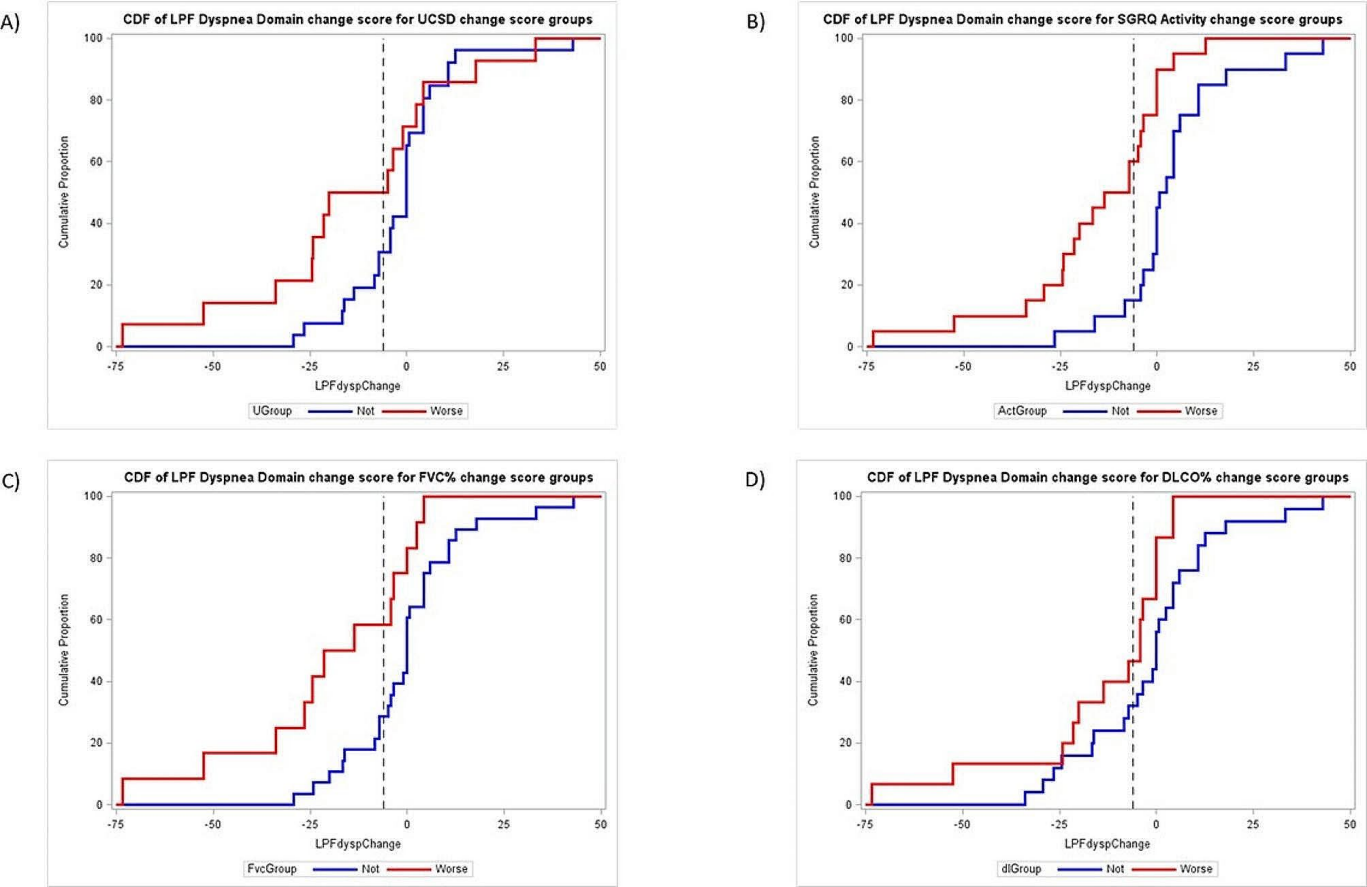
CDF (Cumulative Distribution Function) plots showing baseline-to-week 26 changes in L-PF-35 Dyspnea domain scores for subgroups defined by anchor change, worse or not from baseline to week 26 (Panel A: UCSD anchor, Panel B: SGRQ Activity Domain anchor, Panel C: FVC% anchor, Panel D: DLCO% anchor) values. Footnote: Red = worsened according to anchor; Blue = not worsened (stable/improved) according to anchor; UCSD = University of California San Diego Shortness of Breath Questionnaire; SGRQ = St. George’s Respiratory Questionnaire; FVC% = percentage of the predicted forced vital capacity; DLCO% = percentage of the predicted diffusing capacity of the lung for carbon monoxide; L-PF = 35-item Living with Pulmonary Fibrosis Questionnaire. Definitions for anchors worsened: 1) ≥ 5 point increase for SGRQ Activity domain; 2) ≥ 5 point increase in UCSD score; 3) > 2% drop in FVC% (e.g., 70% to less than 68%); and 4) ≥ 5% drop in DLCO% (e.g., 70–65% or lower)

eCDF plots of L-PF-35 Dyspnea domain 26-week change scores are displayed in Fig. 2. They show separation between subgroups that worsened vs. not at 26 weeks according to each of the four anchors. Table 4 provides values of L-PF-35 Dyspnea domain 26-week change scores for the cohort using percentile cut-points.

**Table 4:** Distribution of L-PF-35 Dyspnea change scores at week 26 for subjects who worsened or not according to each anchor

Anchor	N	Anchor Baseline Value Mean/Median	Difference in L-PF-35 Dyspnea Score from Baseline to 26 Weeks (Baseline minus 26 weeks; negative = worsened)				
			10th Percentile	25th Percentile	50th Percentile	75th Percentile	90th Percentile
UCSD							
-Worse -Not	14 26	52.3 ± 19.2, 53.5 (34.0,74.0) 38.7 ± 28.1, 42.0 (11.0,60.0)	-52.4 -16.7	-24.4 -7.14	-12.5 0.0	2.4 4.2	17.9 10.7
SGRQa							
-Worse -Not	20 20	61.9 ± 22.0, 66.2 (51.3,79.4) 59.4 ± 27.2, 69.9 (44.6,79.7)	-43.2 -12.2	-24.3 -2.2	-10.3 1.5	-1.8 8.3	2.1 25.6
FVC%							
-Worse -Not	12 28	55.9 ± 11.4, 55.5 (50.0,59.0) 63.4 ± 11.5, 64.0 (59.0,67.5)	-52.4 -20.0	-30.2 -7.1	-17.5 0.0	-1.8 5.0	2.4 17.9
DLCO%							
-Worse -Not	15 25	55.2 ± 11.1, 55.0 (48.0,66.0) 51.2 ± 16.8, 48.0 (40.0,58.0)	-52.4 -26.4	-21.4 -8.3	-4.2 0.0	0.0 5.8	4.2 17.6

FVC% = percentage of the predicted forced vital capacity; DLCO% = percentage of the predicted diffusing capacity of the lung for carbon monoxide; L-PF = 35-item Living with Pulmonary Fibrosis Questionnaire; SGRQa = St. George’s Respiratory Questionnaire Activity domain; UCSD = University of California San Diego Shortness of Breath Questionnaire

### MWPC threshold

Predictive modeling yielded estimates for MWPC for worsening in L-PF-35 Dyspnea domain scores of 6.3, 4.8, 8.0 and 6.9 for the four anchors: UCSD, SGRQ Activity, FVC%, and DLCO% respectively. The corresponding point-biserial correlations between L-PF-35 Dyspnea domain score change and the dichotomized UCSD, SGRQ Activity, FVC%, and DLCO% anchors (worse vs. not) were the following: 0.30, 0.49, 0.47, and 0.65. Thus, the weighted MWPC threshold estimate for worsening of L-PF-35 Dyspnea domain scores was 6.6 points (range 5–8).

## Discussion

In this study, we conducted analyses whose results offer a first glance at the psychometric properties of the L-PF-35 Dyspnea domain and support its reliability, validity and the responsiveness of its score as a measure of dyspnea in patients with FHP. Measurement experts and regulatory bodies have compiled criteria that, when met, deem clinical outcome assessments (COAs)– like PROMs– fit for the purpose of measuring outcomes in a target population [10, 18]. The internal structure of the PROM must be sound, with sufficiently strong correlations among grouped items (internal consistency); PROM scores from respondents who are stable on the construct being measured should be similarly stable (test-retest reliability); PROM scores should differ between subgroups of respondents known– or hypothesized– to differ on the construct being measured (known-groups validity); and PROM scores should change for respondents who change on the underlying construct (responsiveness).

Because there are no gold standards for any of the constructs assessed by L-PF-35 scores (including dyspnea), anchors are employed as surrogates for gold standards, and hypotheses are formulated around the surrogates while incorporating the fit-for-purpose criteria outlined above. Anchors, themselves, must be suitable and ideally have undergone validity assessments of their own. Reassuringly, in their studies of patients with PF, other investigators have employed the anchors we used in our analyses [19]. Additionally, self-report anchors (like the UCSD and SGRQ Activity domain) generally surpass expert-endorsed suitability criteria [20], and the FVC and DLCO are universally accepted metrics of PF severity.

As hypothesized, the L-PF-35 Dyspnea domain surpassed the acceptability criteria (0.7) for internal consistency and test-retest reliability. Likewise, L-PF-35 Dyspnea domain scores distinguished respondents hypothesized to have the greatest dyspnea severity (e.g., those with the highest (worst) UCSD scores, highest (worst) SGRQ Activity scores, lowest FVC% or lowest DLCO%) from those with the least dyspnea severity. L-PF-35 Dyspnea domain change scores correlated with anchor change scores, and longitudinal modeling and eCDF plots further supported the L-PF-35 Dyspnea domain score as responsive to changes in dyspnea severity over time.

When the recall period for a PROM is 24 h, variability can be accommodated by averaging scores over a given time frame (e.g., a week). That was not done in the Pirfenidone in FHP trial. However, reassuringly, despite the difference in recall periods (L-PF-35 Dyspnea domain 24 h, UCSD no timeframe, SGRQ Activity domain three months), correlations between anchor change scores were generally moderately strong, statistically significant and always in the hypothesized directions. These results, and previously published data showing a < 1 point day-to-day variability in scores from the L-IPF Dyspnea domain scores over a 14 day period in 125 patients with IPF [6], provide indirect evidence that a single administration of L-PF-35 at each data collection timepoint/visit will likely suffice. And administration on consecutive days with averaging of scores is unlikely to yield significant differences from single administration.

In a previously published study, using different methodology than us, the MWPC threshold for deterioration in the L-PF-44 Dyspnea domain was estimated at 6–7 points in the INBUILD trial population (which included patients with all forms of PF, including FHP, who had progressed within 24 months of enrollment) [21]. The population in the Pirfenidone in FHP trial was similar to the INBUILD population; in both trials, subjects had to have fibrosis and meet the same progression criteria. In our MWPC analysis, we employed predictive modeling, which is argued to yield the most precise MWPC estimates [16]. We did not include distribution-based estimates, because they fail to capture patients’ perspectives, ignore the concept of “minimal”, and arguably, should not be included at all in MWPC estimates [22, 23]. We used a weighting approach that appropriately incorporated the correlation between the L-PF-35 Dyspnea domain score change and anchor change. Doing so yields a less biased estimate than taking the mean or median of all estimates [17]. Regardless, it is reassuring that our point estimate perfectly aligns with the estimate generated from the INBUILD data.

### Limitations

A lack of suitable anchors were available to conduct analyses for the other L-PF-35 scores, so those must be left for future studies (e.g., there were no cough or fatigue questionnaires included in the trial; SGRQ “total” and L-PF-35 “total” are similar in name but not necessarily in the constructs they capture. The same is true for the L-PF-35 Symptoms module and the SGRQ Symptoms domain). Moving forward, investigators would greatly help advance the science of measurement in the ILD field by including patient global impression (PGI) items for all the constructs being evaluated (e.g., here, these could have included PGI Dyspnea Severity, PGI Cough Severity/Frequency, PGI Fatigue Severity, PGI pulmonary fibrosis-related QOL or PGI general QOL). Additional limitations in our study include the low number of subjects (of predominantly the same ethnic/racial background) and the single-center design of the trial that generated the data, both of which potentially limit generalizing results to the broader FHP population. Because “validation” is not a threshold phenomenon and can not be achieved in a single study, our results should be viewed as only a first– but important– step in the process of confirming L-PF-35 Dyspnea domain scores as fit-for-purpose in this population. Additional research, including validation work, concept elicitation, and cognitive debriefing studies in patients with FHP and other non-IPF populations, is encouraged.

## Conclusions

L-PF-35 Dyspnea domain scores appear to possess acceptable reliability, validity and responsiveness for assessing dyspnea severity in patients with FHP. Additional studies are needed to further support its validity and to assess the psychometric properties of the other five L-PF-35 scores for assessing their respective constructs. For now, it is reasonable to use 5–8 points as the estimated range for the MWPC threshold for worsening for the L-PF-35 Dyspnea domain in patients with FHP.

### Electronic supplementary material

Below is the link to the electronic supplementary material.

Supplementary Material 1

## Data Availability

Data are not publicly available. Parties interested in accessing the data used in this study are encouraged to contact Dr. Fernandez Perez (fernandeze@njhealth.org).
