# Supplementary material for: A first look at the reliability, validity and responsiveness of L-PF-35 dyspnea domain scores in fibrotic hypersensitivity pneumonitis
Source: BMC Pulm Med. 2024 Apr 19;24:188. doi: 10.1186/s12890-024-02991-1 (PMC11031991; doi:10.1186/s12890-024-02991-1)
Supplement: Supplementary file 1 — Supplementary Material 1 [file 12890_2024_2991_MOESM1_ESM.pdf]

Supplementary material

A first look at the reliability, validity and responsiveness of L-PF-35 Dyspnea domain scores in fibrotic hypersensitivity pneumonitis

Hypotheses tested for the L-35 Dyspnea domain scores

The UCSD, SGRQ Activity domain, FVC% and DLCO% were anchors for L-PF-35 Dyspnea domain analyses. For certain analyses, we focused on 26 and 52 weeks, because those are the durations most often employed in fILD trials.

1) Internal consistency at baseline > 0.8 and test-retest reliability at 26 weeks > 0.75 (excellent). 0.7 is the acceptability criterion for both.

2) Pairwise correlations between the L-PF-35 Dyspnea domain and anchor variables ≥ 0.3 at baseline. We expected positive correlations for the UCSD and SGRQ Activity domain and negative correlations for FVC% and DLCO%.

3) At baseline, L-PF-35 Dyspnea domain scores will be significantly associated with the anchors, and subjects in the most severely impaired anchor subgroup would have significantly worse (higher and by at least 1 standard deviation) L-PF-35 Dyspnea scores than subjects in the least severely impaired anchor subgroup.

4) Pairwise correlations between L-PF-35 Dyspnea change and anchor change will be ≥ 0.3 at 26 and 52 weeks.

5) Longitudinal models will show statistically significant associations between L-PF-35 Dyspnea change and anchor change over time.

6) According to eCDF plots, for 6 point worsening in L-PF-35 Dyspnea score at 26, there would be a greater proportion of subjects in the “worse” anchor group compared with the “not worse” anchor group. We used 6 points based on the previously published estimate for the L-PF-44 Dyspnea domain score MWPC.

Table S1. Internal consistency and test-retest coefficients for L-PF scores

Score	Week Base	13	26	39	52	Definition of Stability from Baseline to 26 Weeks for each Anchor	ICC (2,1) 26 Weeks
L-PF Dyspnea domain	0.85	0.85	0.91	0.91	0.91	-5 < ΔUCSD < 5 (N=16)<br>-5 < ΔSGRQ Activity < 5 (N=7)<br>-2 ≤ ΔFVC% ≤ 2 (N=15)<br>-5 < ΔDLCO% < 5 (N=11)	0.86 (0.65, 0.95)<br>0.86 (0.48, 0.98)<br>0.81 (0.53, 0.93)<br>0.88 (0.61, 0.97)

L-PF = Living with Pulmonary Fibrosis; UCSD = University of California San Diego Shortness of Breath Questionnaire; SGRQ = St. George's Respiratory Questionnaire; FVC% = percent predicted forced vital capacity; DLCO% = percent predicted diffusing capacity of the lung for carbon monoxide; ICC (2,1) = intra-class correlation coefficient (two-way mixed-effects model for absolute agreement) for test-retest reliability of L-PF-35 Dyspnea domain scores from baseline to 26 weeks.

Table S2. Pairwise correlations among change scores for L-PF, UCSD, SGRQ, FVC% and DLCO%

	Week	FVC% Δ	DLCO% Δ	UCSDΔ	SGRQact Δ
**LPF Dyspnea Δ**	**13**	-0.38, 0.02	-	0.55, 0.0003	0.27, 0.09
	**26**	-0.53, 0.001	-0.46, 0.006	0.34, 0.03	0.55, 0.0003
	**39**	-0.57, 0.001	-	0.53, 0.0008	0.42, 0.01
	**52**	-0.21, 0.24	-0.46, 0.006	0.49, 0.004	0.37, 0.03

Values = correlation coefficient, p value; FVC% Δ = change in percentage of the predicted forced vital capacity; DLCO% Δ = change in percentage of the predicted diffusing capacity of the lung for carbon monoxide; UCSD Δ = change in University of California San Diego Shortness of Breath Questionnaire; SGRQact Δ = change in SGRQ Activity score

Table S3. Responsiveness of L-PF-35 scores as demonstrated by outcomes of longitudinal models of change in L-PF-35 scores using change in anchor as lone predictor

L-PF-35 score	Anchor	Coefficient ± Standard Error, p value
Dyspnea domain	UCSD	0.63 ± 0.07, <00001
	SGRQact	0.57 ± 0.10, <0.0001
	FVC%	-1.43 ± 0.26, <0.0001
	DLCO%	-1.14 ± 0.18, <0.0001

FVC% = percentage of the predicted forced vital capacity; DLCO% = percentage of the predicted diffusing capacity of the lung for carbon monoxide; L-PF = Living with Pulmonary Fibrosis Questionnaire; SGRQ = St. George's Respiratory Questionnaire Activity domain score; UCSD = University of California San Diego Shortness of Breath Questionnaire; models show the relationship between baseline-to-weeks 13/26/39/52 changes in L-PF-35 Dyspnea domain scores and baseline-to-weeks 13/26/39/52 changes in anchor values
